# Self-Powered Smart Insole for Monitoring Human Gait Signals

**DOI:** 10.3390/s19245336

**Published:** 2019-12-04

**Authors:** Wei Wang, Junyi Cao, Jian Yu, Rong Liu, Chris R. Bowen, Wei-Hsin Liao

**Affiliations:** 1 Key Laboratory of Education Ministry for Modern Design and Rotor-Bearing System, School of Mechanical Engineering, Xi’an Jiaotong University, Xi’an 710049, China; w_wei2013@126.com (W.W.); yujian_05_31@163.com (J.Y.); 2 China Ship Development and Design Center, Wuhan 430064, China; 3 Institute of Textiles and Clothing, The Hong Kong Polytechnic University, Hung Hom, Kowloon, Hong Kong, China; rong.liu@polyu.edu.hk; 4 Materials and Structures Centre, Department of Mechanical Engineering, University of Bath, Bath BA27AY, UK; C.R.Bowen@bath.ac.uk; 5 Department of Mechanical and Automation Engineering, The Chinese University of Hong Kong, Shatin, N.T., Hong Kong, China; whliao@cuhk.edu.hk

**Keywords:** self-powered, piezoelectric, smart insole, gait monitoring, multi-scale entropy

## Abstract

With the rapid development of low-power consumption wireless sensors and wearable electronics, harvesting energy from human motion to enable self-powered sensing is becoming desirable. Herein, a pair of smart insoles integrated with piezoelectric poly(vinylidene fluoride) (PVDF) nanogenerators (NGs) are fabricated to simultaneously harvest energy from human motion and monitor human gait signals. Multi-target magnetron sputtering technology is applied to form the aluminum electrode layers on the surface of the PVDF film and the self-powered insoles are fabricated through advanced 3D seamless flat-bed knitting technology. Output responses of the NGs are measured at different motion speeds and a maximum value of 41 V is obtained, corresponding to an output power of 168.1 μW. By connecting one NG with an external circuit, the influence of external resistance, capacitor, and motion speed on the charging characteristics of the system is systematically investigated. To demonstrate the potential of the smart insoles for monitoring human gait signals, two subjects were asked to walk on a treadmill at different speeds or with a limp. The results show that one can clearly distinguish walking with a limp from regular slow, normal, and fast walking states by using multiscale entropy analysis of the stride intervals.

## 1. Introduction

Over recent decades, the rapid development of low-power consumption wearable electronics such as wireless sensors and health monitoring devices has initiated a new wave of research into small-scale energy supply technologies [1,2,3,4,5]. Although traditional batteries are being developed with smaller size and larger storage capacity, the necessity of frequent replacement and charging process limits their wide-scale application in the field of wearable electronics [6]. To address this problem, the technique of energy harvesting from the thermal and kinetic energy of the human body has become a topic of interest since it provides great potential for the development of longer-lasting or completely self-sustaining power systems [7,8,9]. Previous work has involved a variety of energy harvesters utilizing rigid piezoelectric ceramics [10,11,12] and magnets [13,14,15], which have been investigated extensively. However, the brittle and rigid nature of materials such as piezoelectric ceramics has raised concerns about their flexibility and durability [16,17]. Therefore, an increasing number of energy harvesters are being developed with the property of flexibility and biocompatibility to be better integrated with the wearable and portable electronic devices [18].

In addition to energy harvesting technologies, additional functions have been integrated into wearable electronic devices [4]. This includes healthcare monitoring [8,19,20] since it has the potential to help people, in particular patients and the elders, to monitor their physiological signals to assess health conditions. To date, a variety of flexible nanogenerators (NGs) based on triboelectric [21] and piezoelectric [22] mechanisms have been demonstrated to simultaneously harvest energy and monitor human motion signals. For instance, Pu et al. [16] reported a soft skin-like triboelectric nanogenerator (TENG) that enabled biomechanical energy harvesting and tactile sensing. Shi et al. [23] presented a self-powered smart patch based on the triboelectric effect and electrostatic induction; the device could not only collect energy but also act as a sensor to monitor the motion status of the human body. In addition, Hwang et al. [4] developed a transparent, stretchable, and ultrasensitive strain sensor based on a nanocomposite of AgNW/PEDOT:PSS/PU, and the ability to monitor human activities such as breathing, coughing, and drinking were demonstrated. Although the TENGs have the superiorities of low cost, lightweight nature, and easy fabrication, the need for a relative displacement between the two friction layers will not allow the TENGs to function under continuous excitations [24]. In contrast, flexible piezoelectric nanogenerators (PENGs) using piezoelectric materials such as zinc oxide nanowires, polyvinylidene difluoride (PVDF), and poly(vinylidenefluoride-co-trifluoroethylene) (P(VDF-TrFE)) were extensively investigated due to their relatively large piezoelectric coefficient, *d*_33_, durability, and easy fabrication process. As an example, Lee et al. [7] demonstrated a hybrid-fiber NG with a unique piezoelectric layer composed of zinc oxide nanowires and a PVDF infiltrating polymer, and results show that the device could output different voltages under different arm motion, thus acting as a sensor to monitor the motion of a human arm. In addition, Fuh et al. [20] explored the possibility of making highly flexible and durable piezoelectric harvesters by constructing the 3D piezoelectric PVDF fibers on a paper substrate, and experiments demonstrated that the self-powered device had the potential to identify the various behaviors of human motion from the output signals. Although a number of efforts have been made to improve the output performance of PENGs and monitor the human physiological signals, there is limited work focusing on monitoring human gait signals which have the potential to identify the health conditions of a human. Previously, Zhao et al. [10] described a shoe-embedded piezoelectric energy harvester based on a specially designed sandwich structure for energy harvesting from human locomotion. Xing et al. [25] reported a natural design of TENG for harvesting walking energy, in which the human body and ground served as two electrodes of the triboelectric system. However, these efforts only explored the energy harvesting performance of the proposed devices and the potential to monitor the human gait status through the outputs were neglected.

Therefore, we present in this paper a pair of self-powered smart insoles for monitoring the human gait signals. The functional self-powered nanogenerators (NGs) are formed by sputtering aluminum electrode layers on the surface of PVDF layers and four NGs are knitted into the forefoot and hindfoot positions of a pair of insoles through the technology of advanced 3D seamless flat-bed knitting. The electrical output of the NGs under different speeds of locomotion is investigated and a maximum voltage of 41 V is obtained, with a maximum power of 168.1 μW. Additionally, experiments are carried out to study the influence of external load resistance and capacitor on the charging characteristics of the smart insole. Furthermore, two subjects are asked to walk on a treadmill with different speeds or gaits (such as a limp), and the results illustrate that changes in gait are easily identified using multiscale entropy analysis of the stride intervals.

## 2. Fabrication and Experiments

### 2.1. Fabrication of Flexible PVDF Nanogenerator

A PVDF film with a thickness of 80 μm was used and supplied by Company of Fils, South Korea. In order to obtain the functional piezoelectric membrane, a multi-target magnetron sputtering system was applied to sputter aluminum layers on both sides of the PVDF film to act as electrodes. Before the sputtering process, the PVDF film was ultrasonically cleaned for 5 min by using alcohol to remove any contamination. Thereafter, the film was repeatedly flushed by deionized water and then dried in nitrogen. In the fabrication process, repeated experiments were carried out and the optimum thickness of the aluminum electrode layer was determined to be 200 nm to obtain a uniform and electrically conductive film. To ensure that the film was smooth and uniform, a specially designed fixture was applied in the experiments to keep the film stretched during the sputtering process. In order to improve the durability of the film, a layer of PDMS glue was coated on the surface of the obtained PVDF film and then cured for 1 h at a temperature of 40 °C.

### 2.2. Fabrication of Self-Powered Smart Insole

**Figure 1:**
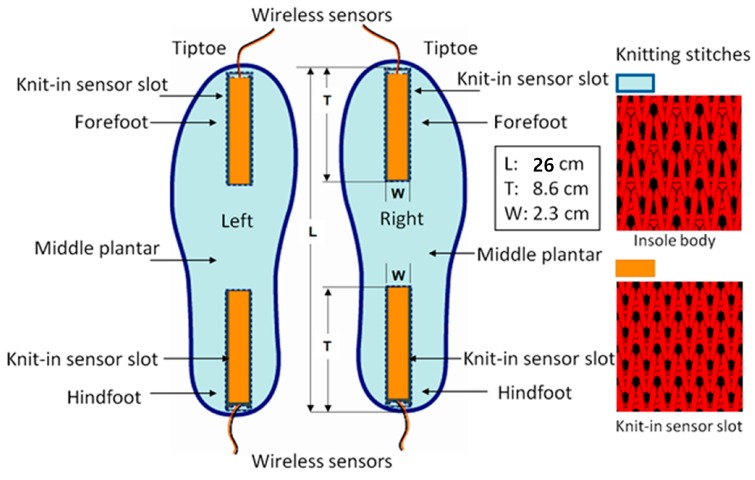
Schematic diagram of the self-powered smart insole.

**Figure 2:**
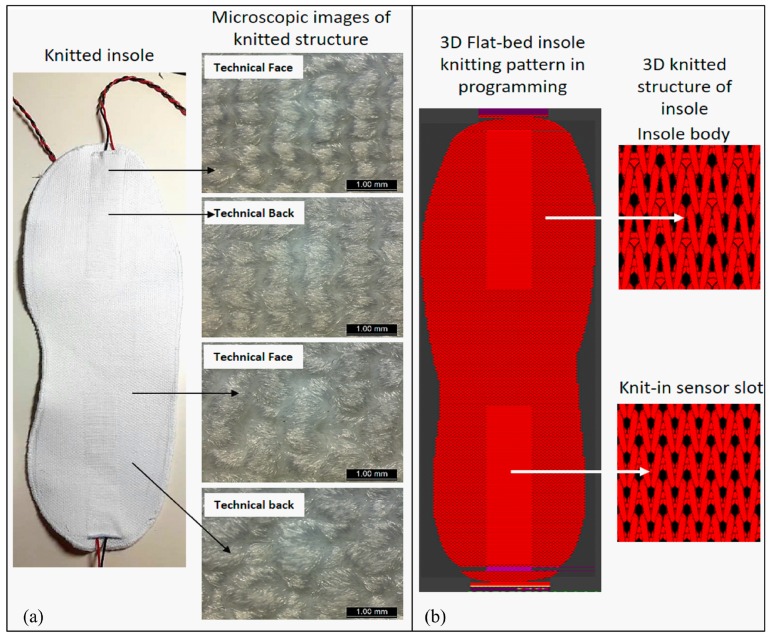
(**a**) Prototype and (**b**) structure of the self-powered smart insole.

The self-powered smart insoles were fabricated by applying advanced 3D seamless flat-bed knitting technology, hybrid multifilament materials, and a layered configuration. To enhance the multi-functional effects of the insole with NGs which also act as sensors, a computer-aided pattern design system (M1plus^®^, Stoll, Germany) was applied to create functional insoles with knit-in sensor slots based on the morphologies of the wearer’s plantar foot with Euro shoe size 42 (26 cm). The functional knitted insole was shaped into three major segments, i.e., forefoot, middle-plantar, and hindfoot, as shown in Figure 1. Two rectangle-shaped knit-in sensor slots (8.6 × 2.3 cm) were set at the forefoot and hindfoot, where the fitting sized NGs were inserted into the knit-in slots to act as sensors to monitor human gait signals. Both knit-in slots and insoles were seamlessly fabricated into one-body via a Stoll computerized flat-bed knitting techniques. Four plies of covered polyamide elastomers with linear density of 40D/40D/34F and two plies of polyester fibers with linear density of 70D/48F/2 were interwoven to form the insole body by deploying specially designed birdseye jacquard knitting structures, which allow the insole to maintain a stable shape when they interact with a plantar foot in dynamic motion. The two seamless knit-in slots were constructed by tubular knitting stitches to leave space for sensor insertion. The fabricated insoles presented good tactile perception, shape retention and air permeability. The average thickness of functional insole is 2.765 mm (±0.004 mm) under compression of 4.9 kPa and the average compression resilience is 53.97% (±0.5%). Figure 2a illustrates the prototype and microscopic knitted structures in technical face and technical back of the smart insole by high-power optical microscope Lacia M165. Figure 2b shows the knitting pattern of insole programmed by Stoll M1 plus system as well as knitting structures of insole body and knit-in sensor slots.

### 2.3. Measurement

Firstly, the fabricated smart insoles were put into a pair of shoes to investigate the energy harvesting performance of the four NGs at the forefoot and hindfoot positions of both feet. Subject A was asked to walk or run on a treadmill at various speeds from 3 km/h to 9 km/h (3~7 km/h for walking and 8~9 km/h for running) to measure the voltage response. The output voltages were measured by an oscilloscope MSOX3052A (Agilent, Wilmington, DE, USA). Furthermore, one of the four NGs was connected with external load resistances and capacitors with different values to investigate the charging characteristic of the system. Finally, two subjects were requested to walk on the treadmill with different speeds or gaits (such as a limp) and the stride intervals obtained from the voltage signals are applied for multiscale entropy analysis to evaluate the status of the gait.

## 3. Results and Discussion

**Figure 3:**
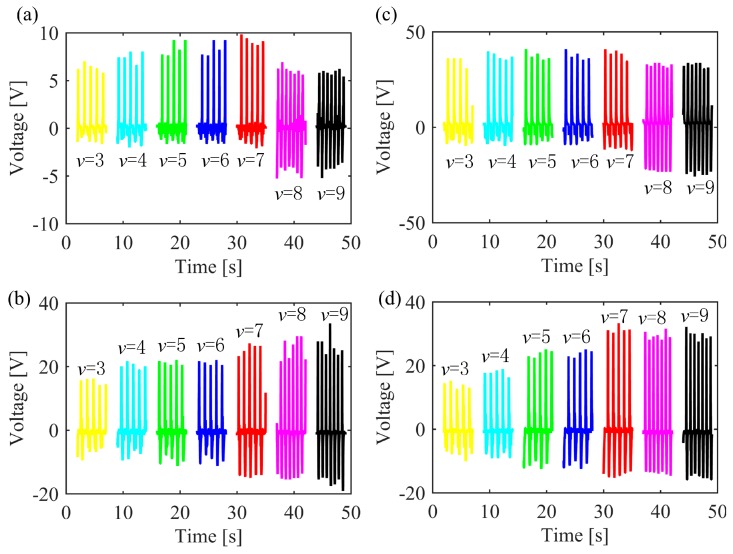
Voltage response of the insole under different motion speed from 3 km/h to 9 km/h: (**a**) left forefoot; (**b**) left hindfoot; (**c**) right forefoot; (**d**) right hindfoot.

Figure 3a illustrates the voltage responses of the NG at the position of left forefoot under different speeds of motion. The output voltages show an increasing trend with an increase in the motion speed from 3 km/h to 7 km/h and the maximum open-circuit voltage was 9.9 V at the speed of 7 km/h. In addition, it is noted that the voltages at these speeds show significantly asymmetric features, which are with small negative values and large positive values. When the speed of motion is increased to 8 km/h and 9 km/h, the maximum positive values of the voltage decrease while the negative values increases, so that the peak-to-peak values voltages are almost unaffected. For the output of the NG at the position of left hindfoot shown in Figure 3b, both the positive and negative values of the voltages increase with an increase in the speed of motion. The maximum positive voltage is obtained at a speed of 9 km/h with a value of 33.6 V, while the maximum negative value is 19.0 V.

Output voltages for the NG at right forefoot are shown in Figure 3c and have a similar trend with that in Figure 3a. The only difference is that the values of the voltages are much larger and the maximum voltage is about 41 V, corresponding to an instantaneous power of 168.1 μW. Furthermore, the output voltage of the NG at the right hindfoot is shown in Figure 3d and the relationship between voltage and motion speed is similar to that in Figure 3b. From Figure 3, it can be seen that the voltages shown in Figure 3a are smaller than that in others, and the reason for this phenomenon is likely to be due to the outputs in Figure 3a were measured with a lower probe resistance of 1 MΩ while the measuring probe resistance for Figure 3b to Figure 3d was 10 MΩ, leading to less discharge of the piezoelectric due to the high time constant (*RC*) and demonstrates the impact of load resistance on peak voltage; where *C* is the capacitance of the piezoelectric and *R* is the load resistance. From the results above, it is demonstrated that the output voltages show different characteristics for the NG at the position of forefoot and hindfoot and the motion speed influences the output greatly, which may be applied to identify the motion status of a human body.

**Figure 4:**
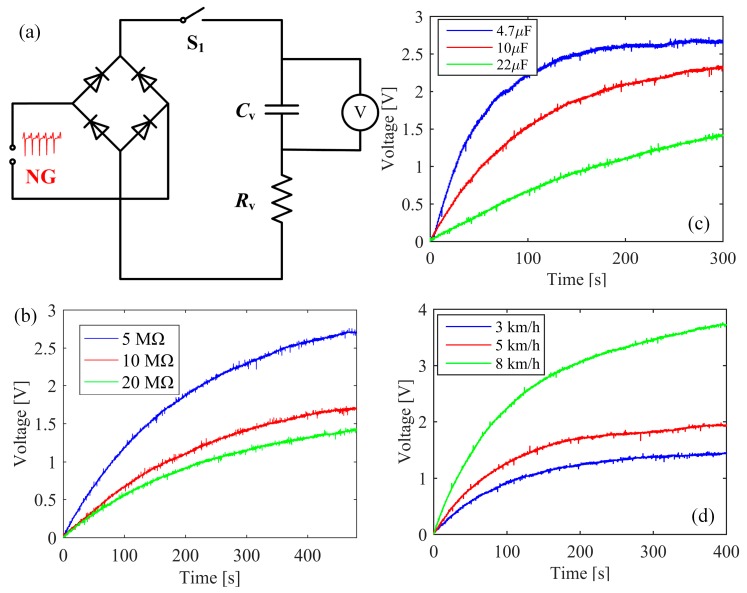
Charging characteristics test of the insole. (**a**) Circuit diagram. (**b**) Charging curve with different values of *R*_v_ and *C*_v_ = 22 μF. (**c**) Charging curve with different values of *C*_v_ and *R*_v_ = 10 MΩ. (**d**) Charging curve with different motion speed when *R*_v_ = 10 MΩ and *C*_v_ = 10 μF.

In order to investigate the influence of external load resistance and capacitor on the charging characteristic of the energy harvesting system, the output signals of the NG at right forefoot was firstly rectified through a bridge rectifier and then connected with external resistance and capacitor, see Figure 4a. The voltage of the capacitor was measured by an oscilloscope MSOX3052A (Agilent, Wilmington, DE, USA) with a probe resistance of 10 MΩ. In the experiments for this investigation, the motion speed of Subject A is 5 km/h which is a comfortable walking speed. To study the influence of the load resistance on the charging characteristic, the applied capacitor is with a capacitance of 22 μF and a rated voltage of 16 V. Under this condition, the charging curves of the capacitor are illustrated in Figure 4b respectively for the connected resistances of 5 MΩ, 10 MΩ, and 20 MΩ. In 500 s, the voltage across the capacitor is 2.7 V when the resistance is 5 MΩ. While the values are 1.7 V and 1.4 V when the resistances are 10 MΩ and 20 MΩ. It is concluded that the charged voltage of the capacitor will decrease with an increase in the value of the connected resistance. On the contrary, a fixed resistance of 10 MΩ is applied to characterize the effect of various capacitances on the charging characteristic. When the capacitances are 4.7 μF, 10 μF, and 22 μF, see Figure 4c, the capacitors are respectively charged to 2.67 V, 2.34 V, and 1.41 V in 300 s, demonstrating that the charged voltage has an inverse relationship with the value of capacitances. When the external resistance is 10 MΩ and the capacitance is 10 μF, the charging curves of the systems are measured at different motion speeds of 3 km/h, 5 km/h, and 8 km/h and the results are shown in Figure 4d. With an increase in the motion speeds, the charged voltages in 400 s also show an increasing trend respectively with the values of 1.43 V, 1.96 V, and 3.73 V.

**Figure 5:**
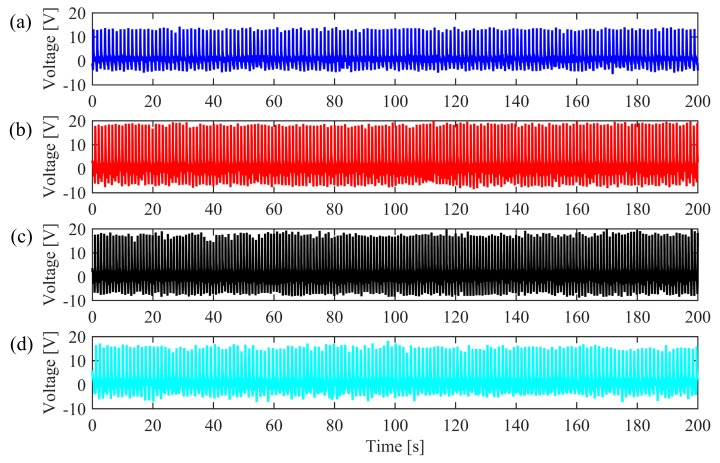
Stability examination. Voltage response of subject A under four motion states for 200 s: (**a**) Walking slow; (**b**) Walking normally; (**c**) Walking fast; (**d**) Walking with a limp.

To demonstrate the potential of the smart insole for human gait signal monitoring, two young, healthy subjects, A and B, were instructed to walk continuously on a treadmill at their self-determined rate of slow, normal and fast speed. Additionally, the subjects were asked to walk with a limp to distinguish from normal data. In the experiments, Subject A was aged 26, with a weight of 70 kg and a height of 179 cm, while Subject B aged 24 was with a weight of 62 kg and a height of 175 cm. The voltage responses under each motion state were measured at least five times for each subject and the outputs of the NG at the position of the right forefoot of Subject A under the four motion states are respectively shown in Figure 5a–d. From the output voltages measured a number of times, it is observed that the output performance of the NG is not influenced, thus the durability of the NG is demonstrated.

**Figure 6:**
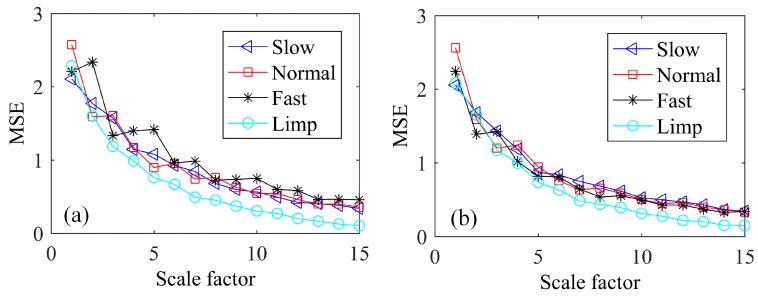
MSE analysis of metronomically paced walking time series obtained from two healthy subjects (**a**) A and (**b**) B who were asked to walk at slow, normal, fast rates and with a limp.

In this paper, stride intervals extracted from the original voltage data of the NG were examined by complexity analysis to identify the status of the human gait. Previously, various evaluation indexes have been used for complexity analysis and have been proposed including multiscale entropy [26], composite multiscale entropy [27], and multivariate multiscale symbolic entropy [28]. In the analysis of entropy, larger values indicate the data is with higher complexity and less regular signals. Herein, to avoid affecting the accuracy due to the length of data points, an overlapping multiscale method [29] was utilized to identify the status of the human gait. The values of the parameters applied to calculate the MSE here are respectively m=2 for embedding dimension, τ=1 for time delay and θ=r×SD for threshold value (r=0.15, SD is the standard deviation of time series). In Figure 6a,b, the calculated MSE of the stride intervals indicates that when the subjects walked at their self-determined rate of slow, normal, fast speed, the complexity of the stride intervals is similar to each other at all scales. However, for walking with a limp, the MSE shows smaller values at larger scales, which indicates a lower complexity or more regular signals. Overall, the values of MSE for all stride interval time series decrease with an increase of scale factor and eventually tend to be stable. In particular, it is demonstrated that one can clearly discriminate walking with a limp from regular slow, normal, fast walking by using the MSE at larger scales. These analyses fully exhibit underlying correlations under different walking conditions, and directly beneficial to health monitoring of human gait.

## 4. Conclusions

In summary, a pair of self-powered smart insoles were presented in this paper for monitoring human gait signals. Multi-target magnetron sputtering system was applied to sputter aluminum electrode layers on the surface of PVDF film to obtain the functional self-powered nanogenerators (NGs). Advanced 3D seamless flat-bed knitting technology, hybrid multifilament materials, and layered configuration were utilized to fabricate the self-powered insoles by knitting the NGs into the position of forefoot and hindfoot of a pair of insoles. Output voltages of the four NGs were measured at different speeds of human motion and a maximum open-circuit voltage of 41 V was obtained, corresponding to a maximum power of 168.1 μW. Then, experiments were carried out to study the influence of external resistance and capacitor on the charging characteristics of the smart insole and results show that the charged voltage of the capacitor decrease with an increase in the value of capacitances and resistances, while it will increase with the increasing of motion speed. Finally, two subjects were asked to walk on a treadmill at different speeds or walking with a limp. Multiscale entropy analysis of the stride intervals clearly identified walking with a limp from regular slow, normal, and fast walking. Finally, the durability of the NG was demonstrated by the collected output voltages many times.
